# Supplementary material for: Ethylene Response Factor LlERF110 Mediates Heat Stress Response via Regulation of LlHsfA3A Expression and Interaction with LlHsfA2 in Lilies (Lilium longiflorum)
Source: Int J Mol Sci. 2022 Dec 17;23(24):16135. doi: 10.3390/ijms232416135 (PMC9781036; doi:10.3390/ijms232416135)
Supplement: Supplementary file 1 [file ijms-23-16135-s001.zip › ijms-2042776-supplementary.pdf]

Supplementary Material

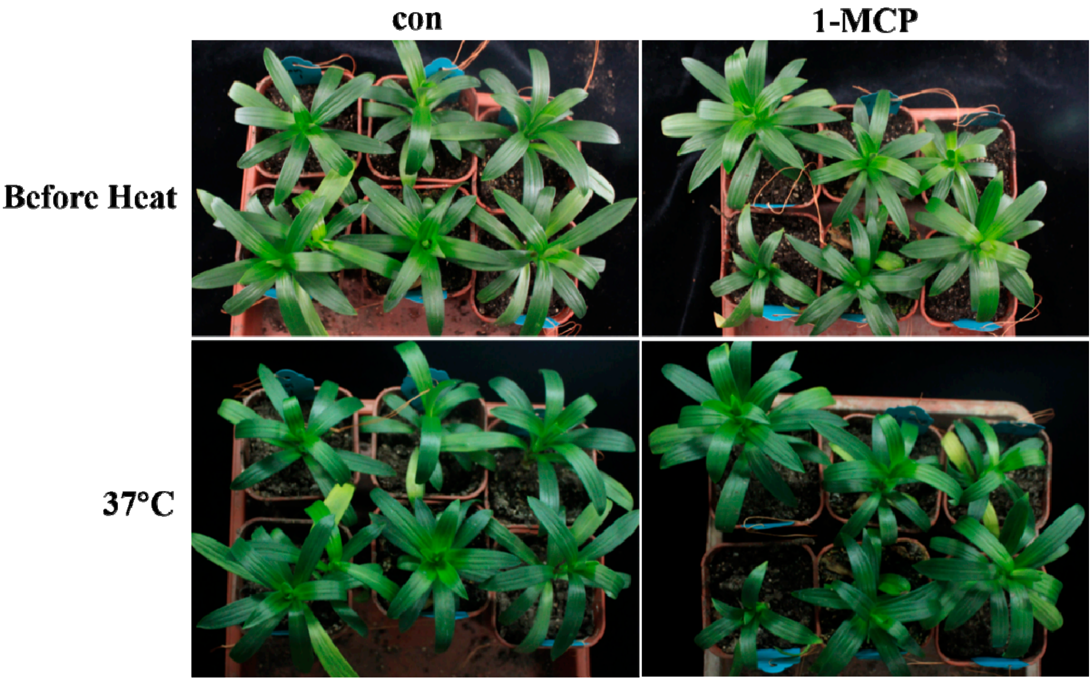

Supplementary Figure S1. Heat stress phenotype of lily plants after 1-MCP (an inhibitor of ethylene receptor) pretreatment. Phenotype of Lily seedlings pretreated with 2 ppm 1-MCP for 3 h and then heat treated at 37 °C for 26 h. There was no difference in phenotype between control and pretreated plants.

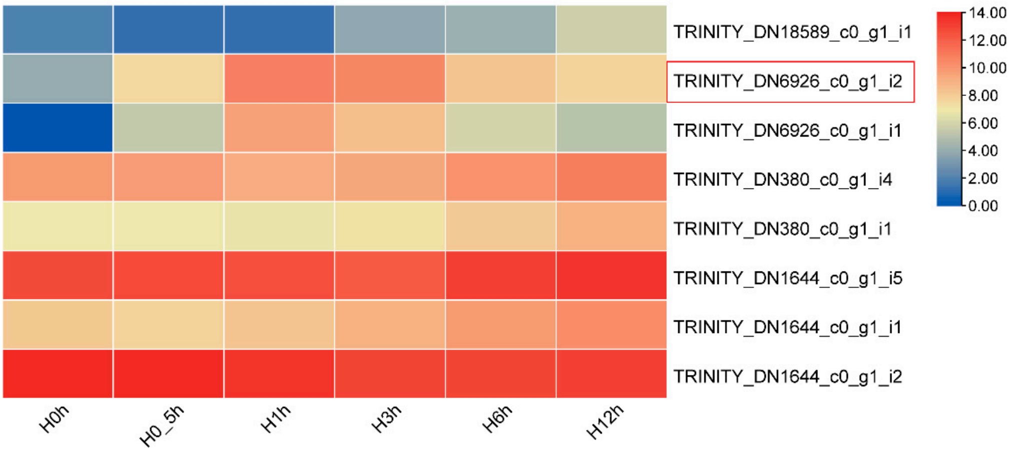

Supplementary Figure S2. Heat map of some *ERF* gene expression in lily leaves with heat treatment. Based on the high-temperature transcriptome of lily, the heat map shows the expression pattern of *ERF* genes with different heat treatment times (0 h, 0.5 h, 1 h, 3 h, 6 h and 12 h), and the red box represents the gene *LlERF110*.

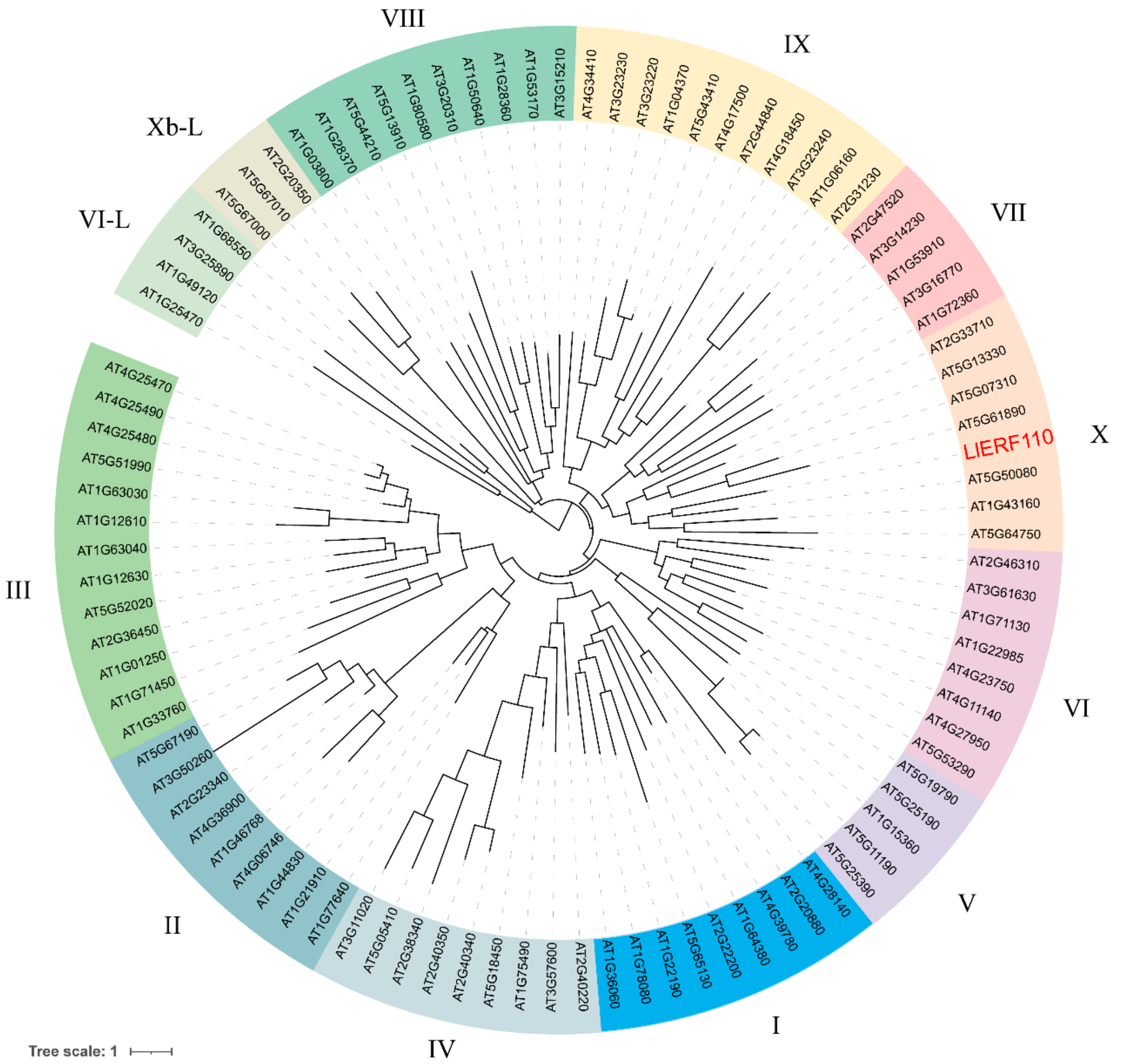

Supplementary Figure S3. Phylogenetic analysis of LlERF110 and AP2/ERF members of Arabidopsis. The amino acid sequences were downloaded from the TAIR website (www.arabidopsis.org) and their AP2 domain was used to construct the tree.The software TBtools was used to reconstruct the evolutionary tree and the iTOL website was used to assembled the tree.

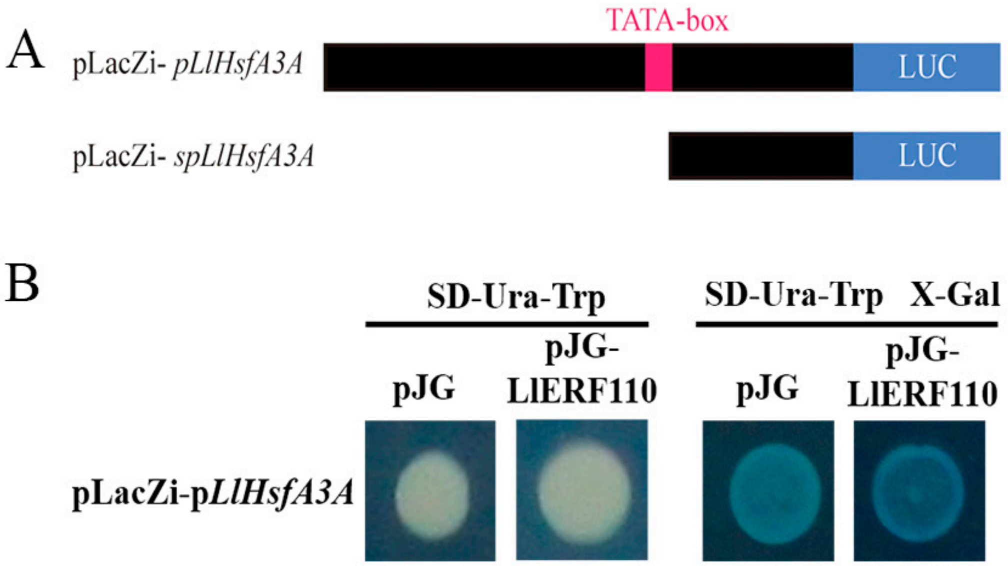

Supplementary Figure S4. Self-activation examination of *LlHsfA3A* promoter in yeast. (**A**) The model of two pLacZi vectors. A 1021bp sequence of the *LlHsfA3A* promoter was cloned into pLacZi to generate pLacZi- *pLlHsfA3A*. A 356 bp sequence of the *LlHsfA3A* promoter without TATA-box was cloned into pLacZi to generate pLacZi- *spLlHsfA3A* (short promoter *LlHsfA3A*). (**B**) Yeast strain Y187 was cotransformed with bait (pLacZi- *pLlHsfA3A*) and preys (pJG or pJG-LlERF110) construct. The bait transformed with the prey pJG were used as negative control. The *LlHsfA3A* promoter has self -activation in yeast. Interaction between bait and prey was determined by cell growth on SD/-Ura-Trp containing X-Gal.

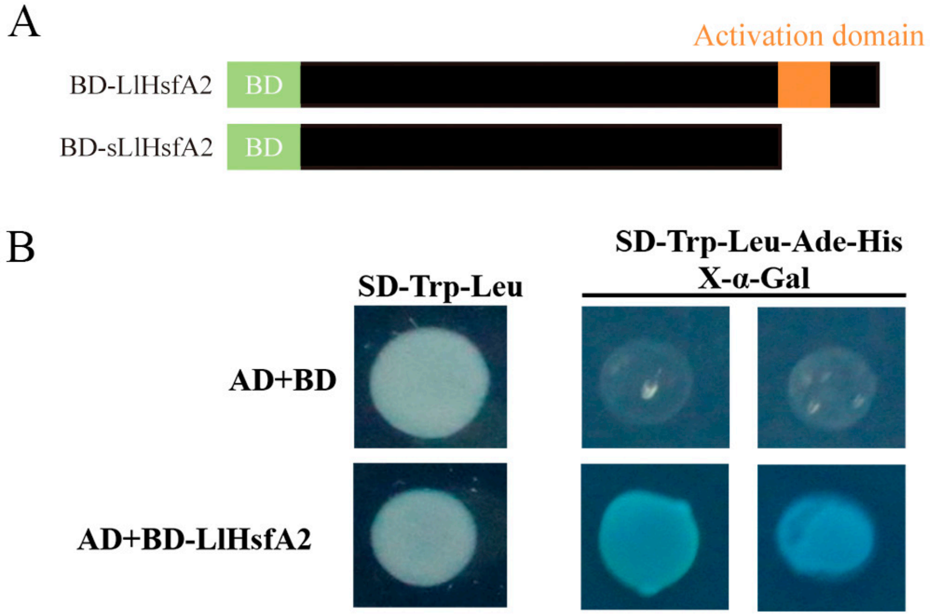

Supplementary Figure S5. Transactivation activity of LlHsfA2 was examined in yeast. (**A**) The

model of two pGBKT7 vectors. A full length sequence of *LlHsfA2* was cloned into pGBKT7 to generate BD-*LlHsfA2*. A sequence of the *LlHsfA2* without activation domain was cloned into pGBKT7 to generate BD-*sLlHsfA2* (short *LlHsfA2*). The empty vector pGADT7(AD) + pGBKT7 (BD) was used as negative control. (**B**) The transformed yeast cells were grown in a SD medium with or without Adenine and histidine. X-α-gal staining was used for detecting β-gal activity.

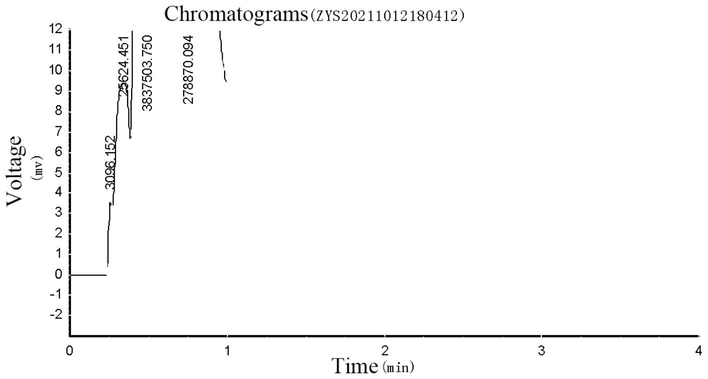

Result table

Peak number	Retention Time	Peak hight	Peak area	Content
1	0.257	2549.239	3096.152	0.0000
2	0.343	4610.413	25624.451	0.0000
3	0.495	516453.906	3837503.750	0.0000
4	0.760	35972.516	278870.094	0.0000
Total		559586.074	4145094.447	0.000

Supplementary Figure S6. Gas Chromatogram of standard ethylene sample. The standard ethylene sample was mas measured by gas chromatograph and exported the chromatogram. The highest peak showed at about 0.5 min, which was the time that ethylene peak showed.

Table S1. Primers used in experiments.

Primer Name	Primer Sequence	Usage
LlERF110-F	5'-ATGTGCTTCAAAGTGGCGACTCCACCAGA-3'	*LlERF110* full-length cDNA clone
LlERF110-R	5'-CTAGCGGGAAGAAGACGGGGGATCCCC-3'	
LlERF110-RT-F	5'-TTGCCGGATAACTGGTGGA-3'	qRT-PCR of *LlERF110*
LlERF110-RT-R	5'-GTGTGCGTTACTTAGCCTGC-3'	
18S rRNA-F	5'-AGTTGGTGGAGCGATTTGTCT-3'	qRT-PCR of *18S rRNA*
18S rRNA-R	5'-CCTGTTATTGCCTCAAACTTCC-3'	
LlHsfA1-F	5'-ATGGGAAGTGTCTATGTGGGG-3'	Primers used for qRT-PCR
LlHsfA1-R	5'-CATTGATACTTGGCAGTTGTTG G-3'	
LlHsfA2-F	5'-GCCAAGGCGGTATTCGAA-3'	
LlHsfA2-R	5'-GCGACAAATCAAACTGACATGG-3'	
LlHsfA3A-F	5'-CTTGGTTTAAGTACGCCAGTGGAAG-3'	
LlHsfA3A-R	5'-GTAAAATATTGTAAAAGAACATGAAGCCTATGG-3'	
LlHsfA4-F	5'-TCTGAGGAGACTGATGTGAATTTG-3'	
LlHsfA4-R	5'-TCTCATGTACCGTCGGCTCTA-3'	
LlHsfA5-F	5'-GTGTCTCCTAGCACGCAGAG-3'	
LlHsfA5-R	5'-TTGGACAGGATGCAAGGGTG-3'	
LlHsp17.6-F	5'-GTCAGCGCTCGTTTCGACTTG-3'	
LlHsp17.6-R	5'-GTGCCAGGTGTCGGTCTTC-3'	
LlHsp22-2-F	5'-CGCTCGCCACTTATCCGTAA-3'	
LlHsp22-2-R	5'-GTTCCGAATCCTCGAGCACA-3'	

LlERF110-2300-F-*Xma*I	5'-GTTACTTCTGCAGGTACCCGGGATGTGCTTCAAAGTGGCG-3'	p35S:: LlERF110-GFP vector construction
LlERF110-2300-R-*Spe*I	5'-TCGACTCTAGAGGATCCCCGGGGCGGGAAGAAGACGGGGGAT-3'	
LlERF110-BD-F-*Nde*I	5'-GGAATTCCATATGATGTGCTTCAAAGTGGCGACTCC-3'	BD-LlERF110 vector construction
LlERF110-BD-R-*Eco*RI	5'-GGAATTCCTAGCGGGAA GAAGACGGGGGATC-3'	
LlERF110-pJG-F-*Eco*RI	5'-GATTATGCCTCTCCCGAATTCATGTGCTTCAAAGTGGCGA-3'	pJG -LlERF110 vector construction
LlERF110-pJG-R-*Xho*I	5'-GCGAAGAAGTCCAAAGCTTCTCGAGCTAGCGGGAAGAAGA-3'	
p*LlHsfA2*-pLacZi-F-*Kpn*I	5'-GGGGTACCAGCATTCTCAAACAACCCTCG-3'	pLacZi- *pLlHsfA2* vector construction
p*LlHsfA2*-pLacZi-R-*Sal*I	5'-ACGCGTCGACTGATTGTGATGGAAGTCCAATTG-3'	
p*LlHsfA3A*-pLacZi-F-*Eco*RI	5'-GGAATTCTCCCATTTTGTTGGATATTCGA-3'	pLacZi- *pLlHsfA3A* vector construction
p*LlHsfA3A*-pLacZi-R-*Xma*I	5'-TCCCCCCGGGTGGTGGATTTGAGGGGG -3'	
sp*LlHsfA3A*-pLacZi-F-*Eco*RI	5'-GGAATTCCAAATCATATCTTTCAAGCTAGAAGTTTC-3'	pLacZi- *spLlHsfA3A* vector construction
sp*LlHsfA3A*-pLacZi-R-*Xma*I	5'-TCCCCCCGGGTGGTGGATTTGAGGGGG-3'	
LlERF110-pBD-F-*Age*I	5'-ACTGTATCGCCGACCGGTATGTGCTTCAAAGTGGCGACT-3'	pBD-*LlERF110* vector construction
LlERF110-pBD-R-*Stu*I	5'-GAAACCAGAGTTAAAGGCCTCTAGCGGGAAGAAGACGGGG-3'	
p*LlHsfA3A*-LUC-F-*Kpn*I	5'-GGGGTACCTCCCATTTTGTTGGATATTCGA-3'	*ProLlHsfA3A*:LUC vector construction
p*LlHsfA3A*-LUC-R-*Xma*I	5'-TCCCCCCGGGTGGTGGATTTGAGGGGG-3'	
LlERF110-SK-F-*Xba*I	5'-GCTCTAGAATGTGCTTCAAAGTGGCGACTCC-3'	Pro*35S:LlERF110* vector construction
LlERF110-SK-R-*Xma*I	5'-TCCCCCCGGGCTAGCGGGAAGAAGACGGGG-3'	
LlERF110-AD-F-*Nde*I	5'-GGAATTCCATATGATGTGCTTCAAAGTGGCGACTCC -3'	AD-*LlERF110* vector construction
LlERF110-AD-R-*Eco*RI	5'-GGAATTCCTAGCGGGAAGAAGACGGGGGATC-3'	
s*LlHsfA2*-BD-F-*Nde*I	5'-GGAATTCCATATGATGGCAAGTGAGATGACGAAG-3'	BD-*sLlHsfA2* vector construction
s*LlHsfA2*-BD-R-*Eco*RI	5'-GGAATTCGATTACACCAAAATCTTGGTCAACC-3'	

LlERF110-YCE-F-*Xba*I	5'-GCTCTAGAATGTGCTTCAAAGTGGCGACTCC-3'	YCE-*LlERF110* vector construction
LlERF110-YCE-R-*Xba*I	5'-TCCCCCCGGGGCGGGAA GAAGACGGGGGATC-3'	
LlHsfA2-YNE-F-*Spe*I	5'-GGACTAGTATGGCAAGTGAGATGACGAA-3'	YNE-*LlHsfA2* vector construction
LlHsfA2-YNE-R-*Kpn*I	5'-GGGGTACCAGGCTGGGAATCTAGATAACCC-3'	
LlERF110-pCaBS-γ-F	5'-AAGGAAGTTTAAGAATGGGAACTTACCGGCTT-3'	*LlERF110*-pCaBS-γ vector construction
LlERF110-pCaBS-γ-R	5'-AACCACCACCACCGTCAACACTAAAACACATTACA-3'	
